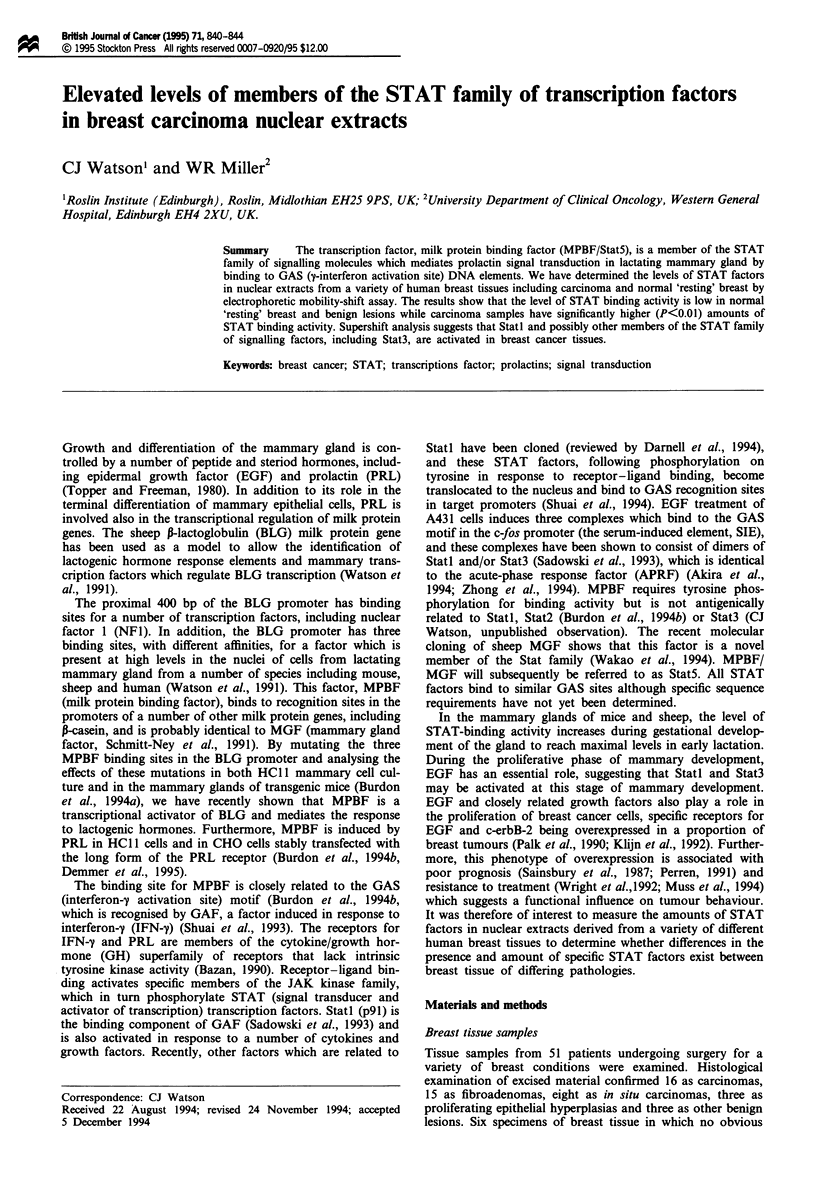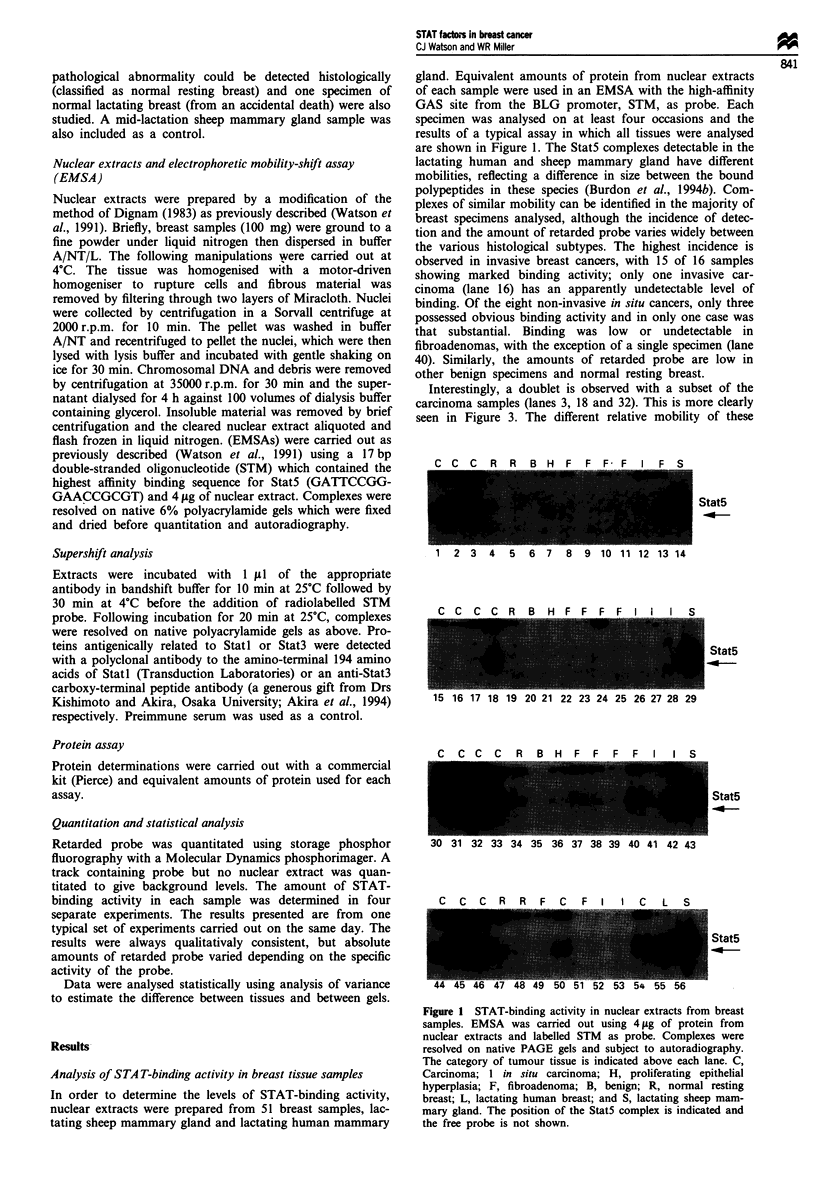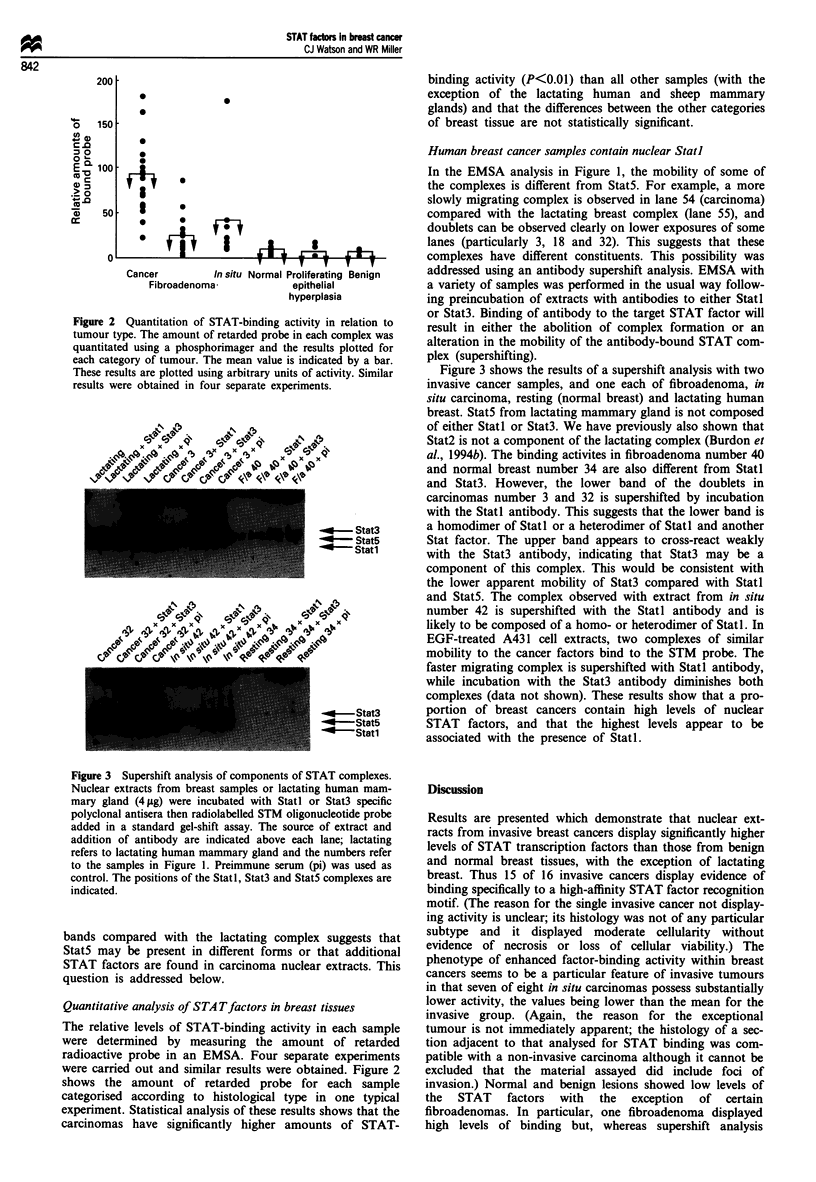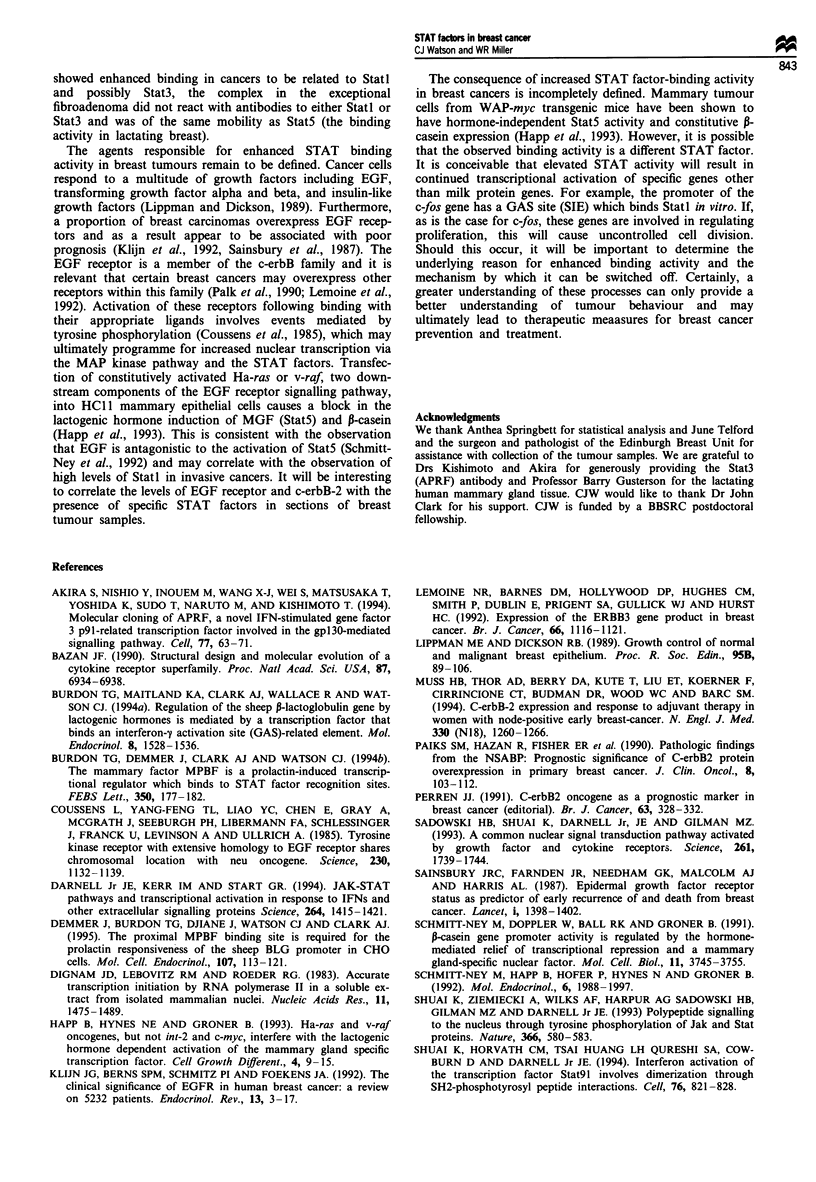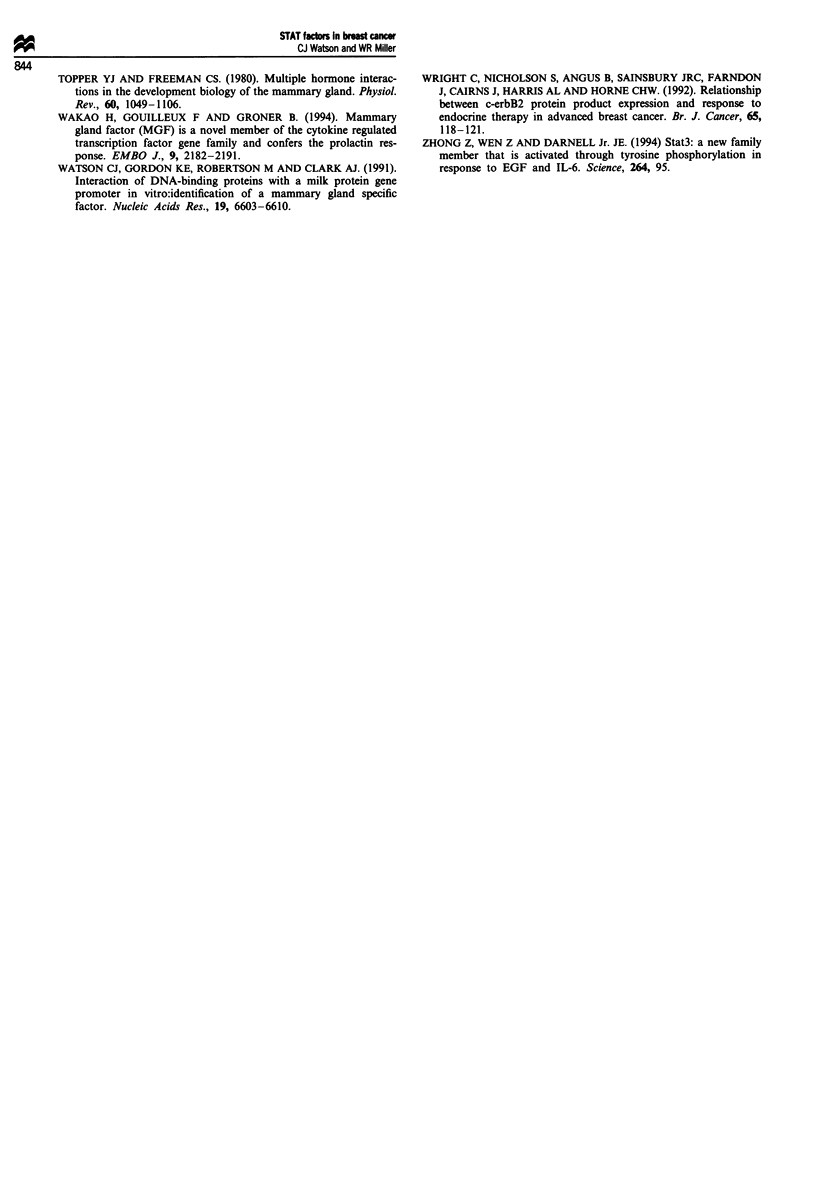# Elevated levels of members of the STAT family of transcription factors in breast carcinoma nuclear extracts.

**DOI:** 10.1038/bjc.1995.162

**Published:** 1995-04

**Authors:** C. J. Watson, W. R. Miller

**Affiliations:** Roslin Institute (Edinburgh), Midlothian, UK.

## Abstract

**Images:**


British Journal of Cancer (1995) 71, 840-844
© 1995 Stockton Press All rights reserved 0007-0920/95 $12.00

### Elevated levels of members of the STAT family of transcription factors in breast carcinoma nuclear extracts

CJ Watson[1] and WR Miller[2]

[1]*Roslin Institute (Edinburgh), Roslin, Midlothian EH25 9PS, UK;* [2]*University Department of Clinical Oncology, Western General Hospital, Edinburgh EH4 2XU, UK.*

**Summary** The transcription factor, milk protein binding factor (MPBF/Stat5), is a member of the STAT family of signalling molecules which mediates prolactin signal transduction in lactating mammary gland by binding to GAS (γ-interferon activation site) DNA elements. We have determined the levels of STAT factors in nuclear extracts from a variety of human breast tissues including carcinoma and normal 'resting' breast by electrophoretic mobility-shift assay. The results show that the level of STAT binding activity is low in normal 'resting' breast and benign lesions while carcinoma samples have significantly higher ($P<0.01$) amounts of STAT binding activity. Supershift analysis suggests that Stat1 and possibly other members of the STAT family of signalling factors, including Stat3, are activated in breast cancer tissues.

**Keywords:** breast cancer; STAT; transcriptions factor; prolactins; signal transduction

Growth and differentiation of the mammary gland is controlled by a number of peptide and steriod hormones, including epidermal growth factor (EGF) and prolactin (PRL) (Topper and Freeman, 1980). In addition to its role in the terminal differentiation of mammary epithelial cells, PRL is involved also in the transcriptional regulation of milk protein genes. The sheep β-lactoglobulin (BLG) milk protein gene has been used as a model to allow the identification of lactogenic hormone response elements and mammary transcription factors which regulate BLG transcription (Watson *et al.*, 1991).

The proximal 400 bp of the BLG promoter has binding sites for a number of transcription factors, including nuclear factor 1 (NF1). In addition, the BLG promoter has three binding sites, with different affinities, for a factor which is present at high levels in the nuclei of cells from lactating mammary gland from a number of species including mouse, sheep and human (Watson *et al.*, 1991). This factor, MPBF (milk protein binding factor), binds to recognition sites in the promoters of a number of other milk protein genes, including β-casein, and is probably identical to MGF (mammary gland factor, Schmitt-Ney *et al.*, 1991). By mutating the three MPBF binding sites in the BLG promoter and analysing the effects of these mutations in both HC11 mammary cell culture and in the mammary glands of transgenic mice (Burdon *et al.*, 1994*a*), we have recently shown that MPBF is a transcriptional activator of BLG and mediates the response to lactogenic hormones. Furthermore, MPBF is induced by PRL in HC11 cells and in CHO cells stably transfected with the long form of the PRL receptor (Burdon *et al.*, 1994*b*, Demmer *et al.*, 1995).

The binding site for MPBF is closely related to the GAS (interferon-γ activation site) motif (Burdon *et al.*, 1994*b*, which is recognised by GAF, a factor induced in response to interferon-γ (IFN-γ) (Shuai *et al.*, 1993). The receptors for IFN-γ and PRL are members of the cytokine/growth hormone (GH) superfamily of receptors that lack intrinsic tyrosine kinase activity (Bazan, 1990). Receptor-ligand binding activates specific members of the JAK kinase family, which in turn phosphorylate STAT (signal transducer and activator of transcription) transcription factors. Stat1 (p91) is the binding component of GAF (Sadowski *et al.*, 1993) and is also activated in response to a number of cytokines and growth factors. Recently, other factors which are related to Stat1 have been cloned (reviewed by Darnell *et al.*, 1994), and these STAT factors, following phosphorylation on tyrosine in response to receptor-ligand binding, become translocated to the nucleus and bind to GAS recognition sites in target promoters (Shuai *et al.*, 1994). EGF treatment of A431 cells induces three complexes which bind to the GAS motif in the c-*fos* promoter (the serum-induced element, SIE), and these complexes have been shown to consist of dimers of Stat1 and/or Stat3 (Sadowski *et al.*, 1993), which is identical to the acute-phase response factor (APRF) (Akira *et al.*, 1994; Zhong *et al.*, 1994). MPBF requires tyrosine phosphorylation for binding activity but is not antigenically related to Stat1, Stat2 (Burdon *et al.*, 1994*b*) or Stat3 (CJ Watson, unpublished observation). The recent molecular cloning of sheep MGF shows that this factor is a novel member of the Stat family (Wakao *et al.*, 1994). MPBF/MGF will subsequently be referred to as Stat5. All STAT factors bind to similar GAS sites although specific sequence requirements have not yet been determined.

In the mammary glands of mice and sheep, the level of STAT-binding activity increases during gestational development of the gland to reach maximal levels in early lactation. During the proliferative phase of mammary development, EGF has an essential role, suggesting that Stat1 and Stat3 may be activated at this stage of mammary development. EGF and closely related growth factors also play a role in the proliferation of breast cancer cells, specific receptors for EGF and c-erbB-2 being overexpressed in a proportion of breast tumours (Palk *et al.*, 1990; Klijn *et al.*, 1992). Furthermore, this phenotype of overexpression is associated with poor prognosis (Sainsbury *et al.*, 1987; Perren, 1991) and resistance to treatment (Wright *et al.*,1992; Muss *et al.*, 1994) which suggests a functional influence on tumour behaviour. It was therefore of interest to measure the amounts of STAT factors in nuclear extracts derived from a variety of different human breast tissues to determine whether differences in the presence and amount of specific STAT factors exist between breast tissue of differing pathologies.

### Materials and methods

### *Breast tissue samples*

Tissue samples from 51 patients undergoing surgery for a variety of breast conditions were examined. Histological examination of excised material confirmed 16 as carcinomas, 15 as fibroadenomas, eight as *in situ* carcinomas, three as proliferating epithelial hyperplasias and three as other benign lesions. Six specimens of breast tissue in which no obvious

Correspondence: CJ Watson
Received 22 August 1994; revised 24 November 1994; accepted 5 December 1994

pathological abnormality could be detected histologically (classified as normal resting breast) and one specimen of normal lactating breast (from an accidental death) were also studied. A mid-lactation sheep mammary gland sample was also included as a control.

*Nuclear extracts and electrophoretic mobility-shift assay (EMSA)*

Nuclear extracts were prepared by a modification of the method of Dignam (1983) as previously described (Watson *et al.*, 1991). Briefly, breast samples (100 mg) were ground to a fine powder under liquid nitrogen then dispersed in buffer A/NT/L. The following manipulations were carried out at 4°C. The tissue was homogenised with a motor-driven homogeniser to rupture cells and fibrous material was removed by filtering through two layers of Miracloth. Nuclei were collected by centrifugation in a Sorvall centrifuge at 2000 r.p.m. for 10 min. The pellet was washed in buffer A/NT and recentrifuged to pellet the nuclei, which were then lysed with lysis buffer and incubated with gentle shaking on ice for 30 min. Chromosomal DNA and debris were removed by centrifugation at 35000 r.p.m. for 30 min and the supernatant dialysed for 4 h against 100 volumes of dialysis buffer containing glycerol. Insoluble material was removed by brief centrifugation and the cleared nuclear extract aliquoted and flash frozen in liquid nitrogen. (EMSAs) were carried out as previously described (Watson *et al.*, 1991) using a 17 bp double-stranded oligonucleotide (STM) which contained the highest affinity binding sequence for Stat5 (GATTCCGG-GAACCGCGT) and 4 μg of nuclear extract. Complexes were resolved on native 6% polyacrylamide gels which were fixed and dried before quantitation and autoradiography.

*Supershift analysis*

Extracts were incubated with 1 μl of the appropriate antibody in bandshift buffer for 10 min at 25°C followed by 30 min at 4°C before the addition of radiolabelled STM probe. Following incubation for 20 min at 25°C, complexes were resolved on native polyacrylamide gels as above. Proteins antigenically related to Stat1 or Stat3 were detected with a polyclonal antibody to the amino-terminal 194 amino acids of Stat1 (Transduction Laboratories) or an anti-Stat3 carboxy-terminal peptide antibody (a generous gift from Drs Kishimoto and Akira, Osaka University; Akira *et al.*, 1994) respectively. Preimmune serum was used as a control.

*Protein assay*

Protein determinations were carried out with a commercial kit (Pierce) and equivalent amounts of protein used for each assay.

*Quantitation and statistical analysis*

Retarded probe was quantitated using storage phosphor fluorography with a Molecular Dynamics phosphorimager. A track containing probe but no nuclear extract was quantitated to give background levels. The amount of STAT-binding activity in each sample was determined in four separate experiments. The results presented are from one typical set of experiments carried out on the same day. The results were always qualitativaly consistent, but absolute amounts of retarded probe varied depending on the specific activity of the probe.

Data were analysed statistically using analysis of variance to estimate the difference between tissues and between gels.

### Results

*Analysis of STAT-binding activity in breast tissue samples*

In order to determine the levels of STAT-binding activity, nuclear extracts were prepared from 51 breast samples, lactating sheep mammary gland and lactating human mammary gland. Equivalent amounts of protein from nuclear extracts of each sample were used in an EMSA with the high-affinity GAS site from the BLG promoter, STM, as probe. Each specimen was analysed on at least four occasions and the results of a typical assay in which all tissues were analysed are shown in Figure 1. The Stat5 complexes detectable in the lactating human and sheep mammary gland have different mobilities, reflecting a difference in size between the bound polypeptides in these species (Burdon *et al.*, 1994*b*). Complexes of similar mobility can be identified in the majority of breast specimens analysed, although the incidence of detection and the amount of retarded probe varies widely between the various histological subtypes. The highest incidence is observed in invasive breast cancers, with 15 of 16 samples showing marked binding activity; only one invasive carcinoma (lane 16) has an apparently undetectable level of binding. Of the eight non-invasive *in situ* cancers, only three possessed obvious binding activity and in only one case was that substantial. Binding was low or undetectable in fibroadenomas, with the exception of a single specimen (lane 40). Similarly, the amounts of retarded probe are low in other benign specimens and normal resting breast.

Interestingly, a doublet is observed with a subset of the carcinoma samples (lanes 3, 18 and 32). This is more clearly seen in Figure 3. The different relative mobility of these

**Figure 1** STAT-binding activity in nuclear extracts from breast samples. EMSA was carried out using 4 μg of protein from nuclear extracts and labelled STM as probe. Complexes were resolved on native PAGE gels and subject to autoradiography. The category of tumour tissue is indicated above each lane. C, Carcinoma; I *in situ* carcinoma; H, proliferating epithelial hyperplasia; F, fibroadenoma; B, benign; R, normal resting breast; L, lactating human breast; and S, lactating sheep mammary gland. The position of the Stat5 complex is indicated and the free probe is not shown.

**Figure 2** Quantitation of STAT-binding activity in relation to tumour type. The amount of retarded probe in each complex was quantitated using a phosphorimager and the results plotted for each category of tumour. The mean value is indicated by a bar. These results are plotted using arbitrary units of activity. Similar results were obtained in four separate experiments.

**Figure 3** Supershift analysis of components of STAT complexes. Nuclear extracts from breast samples or lactating human mammary gland (4 μg) were incubated with Stat1 or Stat3 specific polyclonal antisera then radiolabelled STM oligonucleotide probe added in a standard gel-shift assay. The source of extract and addition of antibody are indicated above each lane; lactating refers to lactating human mammary gland and the numbers refer to the samples in Figure 1. Preimmune serum (pi) was used as control. The positions of the Stat1, Stat3 and Stat5 complexes are indicated.

bands compared with the lactating complex suggests that Stat5 may be present in different forms or that additional STAT factors are found in carcinoma nuclear extracts. This question is addressed below.

*Quantitative analysis of STAT factors in breast tissues*

The relative levels of STAT-binding activity in each sample were determined by measuring the amount of retarded radioactive probe in an EMSA. Four separate experiments were carried out and similar results were obtained. Figure 2 shows the amount of retarded probe for each sample categorised according to histological type in one typical experiment. Statistical analysis of these results shows that the carcinomas have significantly higher amounts of STAT-binding activity ($P<0.01$) than all other samples (with the exception of the lactating human and sheep mammary glands) and that the differences between the other categories of breast tissue are not statistically significant.

*Human breast cancer samples contain nuclear Stat1*

In the EMSA analysis in Figure 1, the mobility of some of the complexes is different from Stat5. For example, a more slowly migrating complex is observed in lane 54 (carcinoma) compared with the lactating breast complex (lane 55), and doublets can be observed clearly on lower exposures of some lanes (particularly 3, 18 and 32). This suggests that these complexes have different constituents. This possibility was addressed using an antibody supershift analysis. EMSA with a variety of samples was performed in the usual way following preincubation of extracts with antibodies to either Stat1 or Stat3. Binding of antibody to the target STAT factor will result in either the abolition of complex formation or an alteration in the mobility of the antibody-bound STAT complex (supershifting).

Figure 3 shows the results of a supershift analysis with two invasive cancer samples, and one each of fibroadenoma, *in situ* carcinoma, resting (normal breast) and lactating human breast. Stat5 from lactating mammary gland is not composed of either Stat1 or Stat3. We have previously also shown that Stat2 is not a component of the lactating complex (Burdon *et al.*, 1994*b*). The binding activites in fibroadenoma number 40 and normal breast number 34 are also different from Stat1 and Stat3. However, the lower band of the doublets in carcinomas number 3 and 32 is supershifted by incubation with the Stat1 antibody. This suggests that the lower band is a homodimer of Stat1 or a heterodimer of Stat1 and another Stat factor. The upper band appears to cross-react weakly with the Stat3 antibody, indicating that Stat3 may be a component of this complex. This would be consistent with the lower apparent mobility of Stat3 compared with Stat1 and Stat5. The complex observed with extract from *in situ* number 42 is supershifted with the Stat1 antibody and is likely to be composed of a homo- or heterodimer of Stat1. In EGF-treated A431 cell extracts, two complexes of similar mobility to the cancer factors bind to the STM probe. The faster migrating complex is supershifted with Stat1 antibody, while incubation with the Stat3 antibody diminishes both complexes (data not shown). These results show that a proportion of breast cancers contain high levels of nuclear STAT factors, and that the highest levels appear to be associated with the presence of Stat1.

### Discussion

Results are presented which demonstrate that nuclear extracts from invasive breast cancers display significantly higher levels of STAT transcription factors than those from benign and normal breast tissues, with the exception of lactating breast. Thus 15 of 16 invasive cancers display evidence of binding specifically to a high-affinity STAT factor recognition motif. (The reason for the single invasive cancer not displaying activity is unclear; its histology was not of any particular subtype and it displayed moderate cellularity without evidence of necrosis or loss of cellular viability.) The phenotype of enhanced factor-binding activity within breast cancers seems to be a particular feature of invasive tumours in that seven of eight *in situ* carcinomas possess substantially lower activity, the values being lower than the mean for the invasive group. (Again, the reason for the exceptional tumour is not immediately apparent; the histology of a section adjacent to that analysed for STAT binding was compatible with a non-invasive carcinoma although it cannot be excluded that the material assayed did include foci of invasion.) Normal and benign lesions showed low levels of the STAT factors with the exception of certain fibroadenomas. In particular, one fibroadenoma displayed high levels of binding but, whereas supershift analysis showed enhanced binding in cancers to be related to Stat1 and possibly Stat3, the complex in the exceptional fibroadenoma did not react with antibodies to either Stat1 or Stat3 and was of the same mobility as Stat5 (the binding activity in lactating breast).

The agents responsible for enhanced STAT binding activity in breast tumours remain to be defined. Cancer cells respond to a multitude of growth factors including EGF, transforming growth factor alpha and beta, and insulin-like growth factors (Lippman and Dickson, 1989). Furthermore, a proportion of breast carcinomas overexpress EGF receptors and as a result appear to be associated with poor prognosis (Klijn *et al.*, 1992, Sainsbury *et al.*, 1987). The EGF receptor is a member of the c-erbB family and it is relevant that certain breast cancers may overexpress other receptors within this family (Palk *et al.*, 1990; Lemoine *et al.*, 1992). Activation of these receptors following binding with their appropriate ligands involves events mediated by tyrosine phosphorylation (Coussens *et al.*, 1985), which may ultimately programme for increased nuclear transcription via the MAP kinase pathway and the STAT factors. Transfection of constitutively activated Ha-*ras* or v-*raf*, two downstream components of the EGF receptor signalling pathway, into HC11 mammary epithelial cells causes a block in the lactogenic hormone induction of MGF (Stat5) and β-casein (Happ *et al.*, 1993). This is consistent with the observation that EGF is antagonistic to the activation of Stat5 (Schmitt-Ney *et al.*, 1992) and may correlate with the observation of high levels of Stat1 in invasive cancers. It will be interesting to correlate the levels of EGF receptor and c-erbB-2 with the presence of specific STAT factors in sections of breast tumour samples.

The consequence of increased STAT factor-binding activity in breast cancers is incompletely defined. Mammary tumour cells from WAP-*myc* transgenic mice have been shown to have hormone-independent Stat5 activity and constitutive β-casein expression (Happ *et al.*, 1993). However, it is possible that the observed binding activity is a different STAT factor. It is conceivable that elevated STAT activity will result in continued transcriptional activation of specific genes other than milk protein genes. For example, the promoter of the c-*fos* gene has a GAS site (SIE) which binds Stat1 *in vitro*. If, as is the case for c-*fos*, these genes are involved in regulating proliferation, this will cause uncontrolled cell division. Should this occur, it will be important to determine the underlying reason for enhanced binding activity and the mechanism by which it can be switched off. Certainly, a greater understanding of these processes can only provide a better understanding of tumour behaviour and may ultimately lead to therapeutic meaasures for breast cancer prevention and treatment.

**Acknowledgments**
We thank Anthea Springbett for statistical analysis and June Telford and the surgeon and pathologist of the Edinburgh Breast Unit for assistance with collection of the tumour samples. We are grateful to Drs Kishimoto and Akira for generously providing the Stat3 (APRF) antibody and Professor Barry Gusterson for the lactating human mammary gland tissue. CJW would like to thank Dr John Clark for his support. CJW is funded by a BBSRC postdoctoral fellowship.

### References

AKIRA S, NISHIO Y, INOUEM M, WANG X-J, WEI S, MATSUSAKA T, YOSHIDA K, SUDO T, NARUTO M, AND KISHIMOTO T. (1994). Molecular cloning of APRF, a novel IFN-stimulated gene factor 3 p91-related transcription factor involved in the gp130-mediated signalling pathway. *Cell*, **77**, 63–71.

BAZAN JF. (1990). Structural design and molecular evolution of a cytokine receptor superfamily. *Proc. Natl Acad. Sci. USA*, **87**, 6934–6938.

BURDON TG, MAITLAND KA, CLARK AJ, WALLACE R AND WATSON CJ. (1994*a*). Regulation of the sheep β-lactoglobulin gene by lactogenic hormones is mediated by a transcription factor that binds an interferon-γ activation site (GAS)-related element. *Mol. Endocrinol.* **8**, 1528–1536.

BURDON TG, DEMMER J, CLARK AJ AND WATSON CJ. (1994*b*). The mammary factor MPBF is a prolactin-induced transcriptional regulator which binds to STAT factor recognition sites. *FEBS Lett.*, **350**, 177–182.

COUSSENS L, YANG-FENG TL, LIAO YC, CHEN E, GRAY A, MCGRATH J, SEEBURGH PH, LIBERMANN FA, SCHLESSINGER J, FRANCK U, LEVINSON A AND ULLRICH A. (1985). Tyrosine kinase receptor with extensive homology to EGF receptor shares chromosomal location with neu oncogene. *Science*, **230**, 1132–1139.

DARNELL Jr JE, KERR IM AND START GR. (1994). JAK-STAT pathways and transcriptional activation in response to IFNs and other extracellular signalling proteins *Science*, **264**, 1415–1421.

DEMMER J, BURDON TG, DJIANE J, WATSON CJ AND CLARK AJ. (1995). The proximal MPBF binding site is required for the prolactin responsiveness of the sheep BLG promoter in CHO cells. *Mol. Cell. Endocrinol.*, **107**, 113–121.

DIGNAM JD, LEBOVITZ RM AND ROEDER RG. (1983). Accurate transcription initiation by RNA polymerase II in a soluble extract from isolated mammalian nuclei. *Nucleic Acids Res.*, **11**, 1475–1489.

HAPP B, HYNES NE AND GRONER B. (1993). Ha-*ras* and v-*raf* oncogenes, but not *int*-2 and c-*myc*, interfere with the lactogenic hormone dependent activation of the mammary gland specific transcription factor. *Cell Growth Different.*, **4**, 9–15.

KLIJN JG, BERNS SPM, SCHMITZ PI AND FOEKENS JA. (1992). The clinical significance of EGFR in human breast cancer: a review on 5232 patients. *Endocrinol. Rev.*, **13**, 3–17.

LEMOINE NR, BARNES DM, HOLLYWOOD DP, HUGHES CM, SMITH P, DUBLIN E, PRIGENT SA, GULLICK WJ AND HURST HC. (1992). Expression of the ERBB3 gene product in breast cancer. *Br. J. Cancer*, **66**, 1116–1121.

LIPPMAN ME AND DICKSON RB. (1989). Growth control of normal and malignant breast epithelium. *Proc. R. Soc. Edin.*, **95B**, 89–106.

MUSS HB, THOR AD, BERRY DA, KUTE T, LIU ET, KOERNER F, CIRRINCIONE CT, BUDMAN DR, WOOD WC AND BARC SM. (1994). C-erbB-2 expression and response to adjuvant therapy in women with node-positive early breast-cancer. *N. Engl. J. Med.* **330** (N18), 1260–1266.

PAIKS SM, HAZAN R, FISHER ER *et al.* (1990). Pathologic findings from the NSABP: Prognostic significance of C-erbB2 protein overexpression in primary breast cancer. *J. Clin. Oncol.*, **8**, 103–112.

PERREN JJ. (1991). C-erbB2 oncogene as a prognostic marker in breast cancer (editorial). *Br. J. Cancer*, **63**, 328–332.

SADOWSKI HB, SHUAI K, DARNELL Jr, JE AND GILMAN MZ. (1993). A common nuclear signal transduction pathway activated by growth factor and cytokine receptors. *Science*, **261**, 1739–1744.

SAINSBURY JRC, FARNDEN JR, NEEDHAM GK, MALCOLM AJ AND HARRIS AL. (1987). Epidermal growth factor receptor status as predictor of early recurrence of and death from breast cancer. *Lancet*, **i**, 1398–1402.

SCHMITT-NEY M, DOPPLER W, BALL RK AND GRONER B. (1991). β-casein gene promoter activity is regulated by the hormone-mediated relief of transcriptional repression and a mammary gland-specific nuclear factor. *Mol. Cell. Biol.*, **11**, 3745–3755.

SCHMITT-NEY M, HAPP B, HOFER P, HYNES N AND GRONER B. (1992). *Mol. Endocrinol.*, **6**, 1988–1997.

SHUAI K, ZIEMIECKI A, WILKS AF, HARPUR AG SADOWSKI HB, GILMAN MZ AND DARNELL Jr JE. (1993) Polypeptide signalling to the nucleus through tyrosine phosphorylation of Jak and Stat proteins. *Nature*, **366**, 580–583.

SHUAI K, HORVATH CM, TSAI HUANG LH QURESHI SA, COWBURN D AND DARNELL Jr JE. (1994). Interferon activation of the transcription factor Stat91 involves dimerization through SH2-phosphotyrosyl peptide interactions. *Cell*, **76**, 821–828.

TOPPER YJ AND FREEMAN CS. (1980). Multiple hormone interactions in the development biology of the mammary gland. *Physiol. Rev.*, **60**, 1049–1106.

WAKAO H, GOUILLEUX F AND GRONER B. (1994). Mammary gland factor (MGF) is a novel member of the cytokine regulated transcription factor gene family and confers the prolactin response. *EMBO J.*, **9**, 2182–2191.

WATSON CJ, GORDON KE, ROBERTSON M AND CLARK AJ. (1991). Interaction of DNA-binding proteins with a milk protein gene promoter in vitro:identification of a mammary gland specific factor. *Nucleic Acids Res.*, **19**, 6603–6610.

WRIGHT C, NICHOLSON S, ANGUS B, SAINSBURY JRC, FARNDON J, CAIRNS J, HARRIS AL AND HORNE CHW. (1992). Relationship between c-erbB2 protein product expression and response to endocrine therapy in advanced breast cancer. *Br. J. Cancer*, **65**, 118–121.

ZHONG Z, WEN Z AND DARNELL Jr. JE. (1994) Stat3: a new family member that is activated through tyrosine phosphorylation in response to EGF and IL-6. *Science*, **264**, 95.